# Development of Asymmetric Vection for Radial Expansion or Contraction Motion: Comparison Between School-Age Children and Adults

**DOI:** 10.1177/2041669518761191

**Published:** 2018-03-21

**Authors:** Nobu Shirai, Shuich Endo, Shigehito Tanahashi, Takeharu Seno, Tomoko Imura

**Affiliations:** Department of Psychology, Faculty of Humanities, Niigata University, Japan; Department of Electrical and Information Engineering, Graduate School of Science and Technology, Niigata University, Japan; Department of Biocybernetics, Faculty of Engineering, Niigata University, Japan; Faculty of Design, Kyushu University, Fukuoka, Japan; Research Center for Applied Perceptual Science, Kyushu University, Fukuoka, Japan; Department of Information Systems, Faculty of Information Culture, Niigata University of International and Information Studies, Japan

**Keywords:** development, vection, expansion or contraction, asymmetry

## Abstract

Vection is illusory self-motion elicited by visual stimuli and is more easily induced by radial contraction than expansion flow in adults. The asymmetric feature of vection was reexamined with 18 younger (age: 6–8 years) and 19 older children (age: 9–11 years) and 20 adults. In each experimental trial, participants observed either radial expansion or contraction flow; the latency, cumulative duration, and saturation of vection were measured. The results indicated that the latency for contraction was significantly shorter than that for expansion in all age-groups. In addition, the latency and saturation were significantly shorter and greater, respectively, in the younger or older children compared with the adults, regardless of the flow pattern. These results indicate that the asymmetry in vection for expansion or contraction flow emerges by school age, and that school-age children experience significantly more rapid and stronger vection than adults.

## Introduction

A large-field visual motion pattern is typically observed when we move thorough the environment. This visual motion pattern, called *optic flow*, contributes to the perception and control of the direction in which we are heading during self-motion (cf. Gibson, 1950). Optimal optic flow creates a compelling perception of self-motion, even if the observer’s body is actually static. This illusory perception of self-movement induced by a visual stimulus is known as *vection* (e.g., Brandt, Dichgans, & Koenig, 1973; Palmisano, Allison, Schira, & Barry, 2015). Broad empirical knowledge regarding vection has accumulated, and the research field has shown remarkable divergence (cf. Palmisano et al., 2015 for a review).

Various motor responses, which may relate to the control of self-motion, are induced by optic flow in very young individuals; infants show postural compensation in response to optic flow (e.g., Lee & Aronson, 1974), and even 3-day-old neonates show similar compensatory patterns in head movements (Jouen, Lepecq, Gapenne, & Bertenthal, 2000). However, previous research has shown that vection (the subjective experience of self-motion induced by visual stimuli) seems to change over time. Lepecq, Giannopulu, and Baudonniere (1995) showed that 7- to 11-year-old children experienced vection under experimental conditions. More recently, Shirai, Seno, and Morohashi (2012) indicated that early school-age children (6–12 year olds) reported vection with significantly shorter latency and stronger saturation than adults, even when the children and adults observed the same visual displays. Similar results were obtained in slightly older children. Shirai, Imura, Tamura, and Seno (2014) tested children in junior high school (13–15 year olds) and found that they experienced significantly stronger vection than did adults. The fact that school-age children tend to experience more rapid or stronger vection than adults do suggests that the developmental change in vection continues beyond childhood.

Although the latency or saturation of vection changes significantly from childhood to adulthood, it is unclear whether other aspects of vection follow a similar developmental trend. Previous studies reported that the experience of vection can be modulated significantly by specific changes in visual stimuli, such as changes in direction of the stimuli. For instance, vection is typically more easily induced by radially contracting visual motion than by expanding motion (e.g., Andersen, 1986; Apthorp, Nagle, & Palmisano, 2014; Berthoz, Pavard, & Young, 1975; Bubka, Bonato, & Palmisano, 2008; Ito & Shibata, 2005; Seno, Ito, Sunaga, & Nakamura, 2010) and by upward than by downward motion (Ito & Takano, 2004; Seya, Shinoda, & Nakaura, 2015). In addition, horizontal translational (rightward and leftward) vection is weaker than vertical (upward and downward) vection (Ito, 2004; Ito & Fujimoto, 2003; Telford & Frost, 1993; Trutoiu, Mohler, Schulte-Pelkum, & Bülthoff, 2009). That is, changes in the direction of visual motion patterns can alter the vection experience, even if other physical properties of visual motion patterns, such as mean speed or pattern size, are constant. It is unknown whether such asymmetries in vection are also observed during childhood; therefore, investigating the development of such asymmetries in vection is an important task to aid in the understanding of the entire developmental process of vection.

The primary aim of the current study was to examine whether asymmetry in vection with a radial expansion or contraction visual motion pattern is observed in school-age children. In addition, we sought to replicate the asymmetry of the expansion or contraction flow in vection. Inconsistent results regarding expansion or contraction asymmetry in vection have been reported. Although many studies have reported the advantage of contraction flow (e.g., Andersen, 1986; Apthorp et al., 2014; Berthoz et al., 1975; Bubka et al., 2008; Ito & Shibata, 2005; Seno et al., 2010), others have reported an advantage of expansion flow (Reinhardt-Rutland, 1982) or no significant asymmetry between expansion and contraction flows (Palmisano et al., 2009).

## Methods

### Ethics Statement

The current study was approved by the Ethics Committee for Psychological Research at Niigata University. All experiments were conducted in accordance with the principles of the Declaration of Helsinki.

### Participants

We included three age-groups: younger children (6–8 years old), older children (9–11 years old), and adults (20–22 years old). We initially tested 60 participants (20 in each age-group) and screened out several participants that were extreme outliers according to the following criterion: participants with a mean individual value greater (or smaller) than the mean ± 3 standard deviations (*SD*) of their age-group for any of the three measurements (latency, duration, or saturation; see the Procedure section). As a result, two younger children and one older child were excluded from the final analysis (for details, see online data https://nyu.databrary.org/volume/468). No participants in the adult group met the exclusion criterion. The final sample was composed of data obtained from 18 younger children (11 females, mean age = 7.84 years, *SD* = 0.90, range = 6.09–8.90 years), 19 older children (8 females, mean age = 10.48 years, *SD* = 0.96, range = 9.12–11.89 years), and 20 adults (8 females, mean age = 21.38 years, *SD* = 0.59, range = 20.08–22.28 years). All participants were healthy and had normal or corrected-to-normal vision; none had a history of visual or vestibular system disease.

### Apparatus and Stimuli

The experiment was conducted in a dark chamber (width = 1.8 m, height = 1.8 m, depth = 5.4 m). Visual stimuli were generated by a computer (MB543J/A; Apple, Cupertino, CA, USA) displayed on a 50-in. video screen (Vsync = 60 Hz, resolution =1024 × 768 pixels) by a digital light-processing projector (MX511; BenQ America Corp., Costa Mesa, CA, USA). The computer was placed outside the experimental chamber and operated by an experimenter during the experiments. The video screen and projector were placed in the chamber. Participants sat on a chair in front of the screen (viewing distance = 57 cm) without any supportive equipment, such as a head or chin rest. The size of the visual stimulus was 102° in width and 76° in height. The visual stimuli were almost identical to those used by Seno et al. (2010) and Shirai et al. (2012) with the exception that not only an expanding optic flow pattern (the same stimulus as that of previous studies) but also a contraction optic flow pattern was used. The expanding (or contracting) optic flow pattern was composed of 16,000 dots randomly positioned inside a simulated cube (20 × 20 × 20 m) in which the virtual viewpoint of an observer moved forward (or backward) at 16 m/s. All stimuli were created using OpenGL (https://www.opengl.org/). On the video screen, 1,240 randomly positioned dots per frame appeared. The dots virtually formed no density gradient. This was partially achieved by reusing dots that moved off the video screen (*dead* dots) to refill areas of the screen with the lowest dot density. Therefore, the number of dots over space-time was maintained constant (i.e., 1,240 dots in a frame). This means that there were fewer dots at greater distances from the simulated viewpoint. In addition, we varied the lifetime of each dot according to its distance from the viewpoint; dots farther from the viewpoint were given shorter lifetimes. Thus, dots that appeared to be at a greater distance in the simulated three-dimensional space tended to cluster around the central area in the projector screen. The shorter lifetime given to more distant dots in the three-dimensional space resulted in the more frequent *death* of dots in the central area of the projector screen and contributed to respawning the *dead* dots in areas with the lowest dot density. Therefore, in theory, there were no static depth cues available from the dot-density gradient, and the only moving depth cue was motion parallax. Each dot was of constant size on the screen and subtended a visual angle of 0.03 to 0.05°, depending on its eccentricity on the screen.

### Procedure

Written informed consent was obtained from all participants (and their parents in the case of children) before starting the experiments. All participants engaged in two experimental conditions: expansion and contraction. Five trials were conducted for each condition; thus, each participant took part in 10 trials. Trials in the expansion and contracting conditions were conducted in an alternating pattern for each participant, and the order of the conditions was counterbalanced across the participants in each age-group. In the expansion (or contraction) condition, each trial consisted of the presentation of an expanding (or contracting) flow pattern lasting for 40 s. The participants were instructed to fixate on the center of the screen during each trial while remaining relaxed. No fixation point was presented.

Three measurements regarding vection (latency, duration, and saturation) were adopted in the current study. Participants were instructed to press one of the assigned buttons on a computer mouse connected to the computer when they perceived self-motion during each trial, so that the latency of vection and the cumulative duration of vection in each trial could be recorded. After each trial, the participants were asked to rate the saturation of vection using a Visual Analogue Scale (VAS; 100 mm in length) printed on paper. The observers drew a short orthogonal line segment on the VAS to estimate the saturation of vection. The distance (in mm) from the left edge of the VAS to the intersection was regarded as the rated saturation; thus, a longer distance indicated higher saturation. All participants were instructed that if they did not feel their body move, they should draw a short vertical line at the left edge of the VAS, while if they felt their body move like real locomotion, they should draw a short line at the right edge of the VAS as a benchmark rating. We did not use any standard stimuli for this estimate of subjective vection saturation, as previous studies (Shirai et al., 2012, 2014) reported that this method is appropriate for children of similar ages to those who participated in the current study.

Two experimenters, the main experimenter (S. E.) and an assistant, were involved in each experiment. The main experimenter was outside the experimental chamber and managed the personal computer, selecting and running the appropriate experimental program for each trial. The assistant stood behind the participant inside the experimental chamber during each experimental trial and supervised the participant’s engagement. At the beginning of each trial, the assistant confirmed that the participant was looking at the projector screen as instructed and was holding the computer mouse. After each trial, the assistant gave the participant a VAS and a pen and then confirmed that the participant marked the analog scale as instructed. All communications between the main experimenter and the participants during experiments were made via the assistant (e.g., if the participant wanted to take a short rest before starting the next trial, she or he asked the assistant, who then asked the main experimenter to delay the next trial). It should be noted that the assistant was naïve regarding the purpose and the hypothesis of the current study. Thus, possible experimenter effects were minimized in the current experimental setting.

## Results

**Figure 1.**
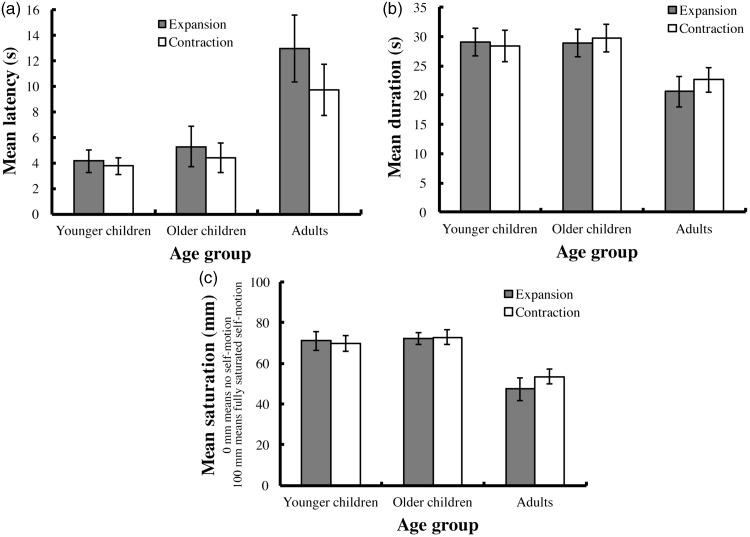
Experimental results. The vertical axes show the (a) mean latency, (b) mean duration of the reported vection, and (c) mean saturation (mean distance between the left edge of a VAS and the vertical intersection drawn by the participants on the scale). Dark bars indicate the results under the expansion condition, and white bars indicate the results under the contraction condition. Error bars indicate ± 1 *SEM*. VAS = visual analogue scale.

The mean latency for vection in each age-group is shown in Figure 1(a). We conducted a two-way mixed analysis of variance (age vs. flow direction) for the latency data and found that the significant main effects of age and flow direction, *F*(2, 54) = 5.978, *p* = .005 and *F*(1, 54) = 6.362, *p* = .015, respectively, while the interaction between age and flow direction was not significant, *F*(2, 54) = 2.163, *p* = .125. Multiple comparisons for the main effect of age using Bonferroni-corrected *t* tests revealed that the mean latency was significantly shorter in the younger and older children than that in the adults, *t*(37) = 3.162, *p* = .008 and *t*(38) = 2.828, *p* = .020, respectively, but was not significantly different between the younger and older children, *t*(36) = 0.369, *p* > .250.

The mean duration of reported vection in each age-group is shown in Figure 1(b). A two-way mixed analysis of variance (age vs. flow direction) indicated that the main effect of age was significant, *F*(2, 54) = 3.196, *p* = .049, while the main effect of flow direction and the interaction were not significant, *F*(1, 54) = 0.984, *p* > .25, *F*(2, 54) = 1.044, *p* > .25, respectively. Multiple comparisons for the main effect of age using Bonferroni-corrected *t* tests found no significant difference in duration among the three age-groups (younger vs. older children: *t*(36) = 0.166, *p* > .250; younger children vs. adults: *t*(37) = 2.100, *p* = .121; older children vs. adults: *t*(38) = 2.300, *p* = .076).

The mean saturation of reported vection in each age-group is shown in Figure 1(c). The main effect of age on saturation was significant, *F*(2, 54) = 10.677, *p* < .001, while the main effect of flow direction and the interaction were not significant, *F*(1, 54) = 0.181, *p* > .250; *F*(2, 54) = 1.208, *p* > .250, respectively. Multiple comparisons for the main effect of age using Bonferroni-corrected *t* tests revealed that mean saturation was significantly greater in the younger and older children than that in the adults, *t*(37) = 4.282, *p* < .001 and *t*(38) = 3.732, *p* = .001, respectively, but there was no significant difference in mean saturation between the younger and older children, *t*(36) = 0.485, *p* > .250.

## Discussion

The present study had two main findings. First, the significant main effect of flow direction and the lack of a significant interaction between age and flow direction in the latency data indicate that the onset of vection was faster under the presentation of contracting optic flow than that of expanding optic flow in all age-groups. That is, the asymmetry of expansion or contraction in vection (e.g., Andersen, 1986; Bubka et al., 2008; Ito & Shibata, 2005; Seno et al., 2010) was replicated even in school-age children. The second finding is that, overall, the children reported significantly shorter or higher latency or saturation than the adults did. We discuss the two main findings in the following sections.

### Possibility of Experimenter Effects in the Current Results

One might be concerned about the possibility that participants’ responses were affected by the experimenters’ expectations in the current study. This would render the current findings mainly a product of artifice related to the experimenter effect (e.g., if the participants were susceptible to the experimenters’ demands, they may have endorsed the asymmetric responses in vection; children, in particular, may have been more susceptible to the experimenters’ demands, and their reports of more rapid or stronger vection than adults may reflect this phenomenon).

It should be noted that, because the assistant who directly communicated with the participants during experiments was naïve regarding the purpose and the hypothesis of the current study, such an experimenter effect would be somewhat reduced under the current experimental setting (for details, see the Procedure section). In addition, a comparison between the current study and a previous study that investigated automatic motor responses to optic flow in children and adults is relevant to this concern. Baumberger, Isableu, and Fluckiger (2004) measured postural sway in response to approaching or receding ground flow patterns in children and adults. They reported that, overall, children showed significantly different postural sway patterns in response to the onset and the offset of the flow patterns than did adults. Specifically, the children showed significantly longer or shorter latency to the onset or offset of the approaching flow than the adults did. Moreover, the children showed asymmetrical developmental trends in the sway latency between the approaching and receding flows. Furthermore, there were significant differences in the sway latencies in response to both the onset and offset of the approaching (but not the receding) flow among both children and adults. Although the current study and that of Baumberger et al. (2004) used different measures (verbally instructed responses vs. automatically generated motor actions), both studies reported similar results: Children and adults responded significantly differently to flow patterns that represented self-motion, and responses to expanding or contracting (or approaching or receding) flows were asymmetric. The similarity between the current results (based on verbally instructed responses) and the results of Baumberger et al. (2004, based on the automatically produced motor actions) implies that both sets of findings reflect a common developmental trend in the mechanisms for processing optic flow and self-motion perception or control in childhood. Thus, although the possibility of experimenter effects on the current results cannot be completely ruled out, it is plausible that the current results mainly reflect the developmental process of the mechanisms related to processing optic flow and the perception or control of self-motion.

### Expansion or Contraction Flow Asymmetry in Vection Observed in Latency Data

In the current study, significant asymmetry in the expansion or contraction flow in vection was found in the latency measure but not in the other two measures, duration and saturation. It is possible that, due to the finding’s reliance on only one of the three measures, the observed asymmetry is not a compelling result. Indeed, several previous studies with adult participants reported significant expansion or contraction asymmetry in vection as measured by more than one of the three measures (e.g., Bubka et al., 2008; Seno et al., 2010).

The fact that the asymmetry was observed only in the latency data in the current study suggests that our experimental procedure, specialized for investigating children’s vection, might be best for tapping expansion or contraction flow asymmetry using latency but not duration and saturation. For instance, because young children are not tolerant of long experimental sessions, we adopted a relatively short presentation time (40 s) for each experimental trial. On the other hand, Seno et al. (2010), who reported longer duration and stronger saturation of vection for a contracting than for an expanding flow, used a longer visual stimulus presentation time (60 s). The presentation time used in the current study may have been long enough to pick up the asymmetry in latency data but too short to produce pronounced differences in duration and saturation between the expansion and contraction flows. Interestingly, another previous study (Bubka et al., 2008) succeeded in finding expansion or contraction asymmetry not only in latency but also in saturation using a much shorter presentation time (30 s) than was used in the current study. This might be due to the use of a more sensitive experimental procedure than ours for estimating vection saturation; participants in Bubka et al. estimated the saturation of vection in real time using an analog slider during each experimental trial, whereas the participants in the current study estimated vection saturation using analog visual scales after each trial. It is plausible that estimating vection saturation in real time by handling an analog slider would offer a more sensitive and direct way for adult participants to perform this task (although young children seem to have more difficulty engaging in such complex tasks than adults do).

Moreover, inconsistency among the three independent measures (latency, duration, and saturation) has often been reported in vection studies; thus, it is believed that the three measures tap into different aspects of the vection experience (cf. Seno et al., 2017). For instance, vection latency is thought to represent the time required to resolve visual–vestibular conflict (i.e., visual information representing motion by the observer vs. vestibular information identifying the observer’s static state; e.g., Palmisano, Allison, Kim, & Bonato, 2011; Weech & Troje, 2017; Zacharias & Young, 1981), or it may reflect the time required to suppress the default visual processing regarding object motion before vection emerges (e.g., Palmisano et al., 2015). Duration is often thought to be an index of the strength or resilience of vection, with longer durations representing stronger and more resilient vection experiences. Although saturation is also thought to be an index of vection strength, it may reflect other aspects of subjective experience (e.g., convincingness, intensity, and realism) and may be more susceptible to experimenter expectations and observer cognitions than are latency and duration (e.g., Palmisano & Chan, 2004).

In summary, although the current experimental procedure, which was mainly designed for children, was useful for examining expansion or contraction asymmetry related to one of the several aspects of vection, namely, latency, the procedure may not be optimal for investigating asymmetry related to the other aspects of vection (i.e., duration and saturation). A young-child-friendly experimental procedure for measuring vection saturation in real time during the presentation of a visual stimulus will be necessary in future investigations.

### Relationship Between Expansion or Contraction Asymmetry in Vection and Flow Sensitivity

In this section, we discuss the possible relationship between the known asymmetric detection sensitivity to expansion or contraction flow and the similar vection asymmetry observed in the current latency data.

Many studies have reported that the human visual system has asymmetric sensitivity to radial expansion or contraction flow, although the reported results are mixed; some studies have indicated an advantage in the detection of expansion flow (e.g., Gilmore, Hou, Pettet, & Norcia, 2007; Lewis & McBeath, 2004; Shirai & Yamaguchi, 2004; Takeuchi, 1997), whereas others have indicated an advantage in the detection of contraction flow (Edwards & Badcock, 1993; Edwards & Ibbotson, 2007). Although the reason for the inconsistent results regarding asymmetric sensitivity to radial expansion or contraction remains unclear, sensitivities to expansion and contraction may be independently adaptive visual functions that contribute to detecting and processing ecologically important information contained in radial optic flows (cf. Shirai, Kanazawa, & Yamaguchi, 2006). For instance, because expansion flow represents an object approaching or an observer’s own forward movement, expansion detection seems to have obvious value, namely, in the possibility of avoiding or intercepting an approaching object or of controlling the direction of self-motion. On the other hand, contraction flow represents an object receding from an observer or the observer’s own backward body movement. The advantage of detecting contraction may be the contribution it affords to guidance of hand movements during reaching (Edwards & Badcock, 1993) or secure adjustment to backward body tilt (Edwards & Ibbotson, 2007). The asymmetry of radial expansion or contraction flow detection has been observed even in the first few months of life (advantage of expansion detection: Shirai & Imura, 2014; Shirai, Kanazawa, & Yamaguchi, 2004, 2008; advantage of contraction detection; Shirai et al., 2006, 2009). Thus, it is plausible that both adults and school-age children have an asymmetric sensitivity to radial expansion or contraction flows.

Presumably, the observed contraction advantage in our latency data may be related to the contraction detection advantage previously reported in both adults (e.g., Edwards & Badcock, 1993; Edwards & Ibbotson, 2007) and very young individuals (Shirai et al., 2006, 2009). Edwards and Ibbotson (2007) suggested that the contraction detection advantage contributes to maintaining body balance during postural sway. It is easier to step forward to compensate for postural sway when our body is tilted forward than to step back when our body is tilted backward. Thus, our visual system should be more sensitive to a contraction flow, which represents backward postural sway, than to an expansion flow, which represents forward sway. The fact that more rapid vection for contraction than for expansion flow was observed in the current study fits well with the perspective of Edwards and Ibbotson (2007): Because backward body movements are more dangerous than forward body movements, backward movement should be detected more rapidly than forward movement.

### Why Did the Children Show More Rapid and Stronger Vection Than the Adults?

The current finding that the children had faster and stronger vection than the adults is consistent with a previous vection study in children of a similar age (Shirai et al., 2012; 6–12 year olds). The consistency between the present and previous studies suggests that school-age children tend to experience stronger vection than adults, and that vection is more easily induced in school-age children than in adults. However, the reason that stronger or more rapid vection is observed at younger age remains unclear. Shirai et al. (2012) inferred that a potential difference in the size of the effective visual field between children and adults might affect their experience of vection. Stronger vection can be induced by a larger sized visual stimulus (e.g., Leibowitz, Post, Rodemer, Wadlington, & Lundy, 1980; Ohmi, Howard, & Landolt, 1987). Because the effective size of the visual field is smaller in school-age children than adults (e.g., Holmes, Cohen, Haith, & Morrison, 1977; Lakowski & Aspinall, 1969), a visual stimulus might be seen as larger in a child’s relatively small visual field than in an adult’s larger visual field, even if the children and adults observe exactly the same visual stimulus; thus, induced vection may be greater for children than for adults, even if the children and adults observe the same visual stimulus.

Alternatively, children of this age may rely more on visual information than adults to perceive movement of their own body. When we move through the environment, we perceive self-movement through vision and other sensory information, such as vestibular data. Several studies have shown that human adults integrate those multisensory inputs to perceive the heading direction during self-motion (e.g., Bertin & Berthoz, 2004; Butler, Smith, Campos, & Bulthoff, 2010; Harris, Jenkin, & Zikovitz, 2000). Presumably, school-age children use less nonvisual information than visual information to estimate self-motion. Evidence suggests that vestibular sensitivity is still immature until later school age; vestibular sensitivity develops slowly through toddlerhood and childhood (3–4 years and 14–15 years), and even 14- to 15-year-old children showed significantly lower vestibular sensitivity than adults (Hirabayashi & Iwasaki, 1995). In addition, Lepecq, Giannopulu, Mertz, and Baudonniere (1999) suggested that individual differences in vestibular sensitivity can affect the vection experience. They found that the latency of reported vection tends to be shorter in observers who have lesser vestibular sensitivity than those who have higher vestibular sensitivity. Taken together, stronger and more rapid vection in school-age children compared with that in adults may stem from lesser vestibular sensitivity in school-age children.

### Potential Limits of the Visual Display Used in the Current Study

It should be noted that, because the viewing distance in the current study was relatively short (57 cm) to ensure that the visual stimulus was large in size, the visual display might have produced some unnatural effects as participants viewed the simulated space. For instance, there were inconsistencies in the depth structure simulated by moving dots, and various depth cues (e.g., binocular disparity, vergence, and accommodation) were available from the surface of the projection screen. In addition, the relatively lower resolution of the images (1024 × 768 pixels) projected on the relatively large projection screen might have produced the “screen door effect,” a phenomenon in which gratings composed of edges of pixels appear in front of a displayed image. Such unnatural features of the visual stimuli might have affected the observers’ perception of the depth structure of the visual scene and of their own movement. Thus, the reported vection in the current study may have been distorted due to the unnatural look of the visual stimuli. Future studies using a larger display and higher pixel resolution with a longer viewing distance will be necessary to examine this issue. Such stimuli will reduce the inconsistencies between the simulated depth structure and the view projected on the screen as well as the screen door effect.

## Concluding Remarks and Future Directions

The current results indicate the presence of the known asymmetry for expansion or contraction of optic flow in vection in school-age children. This suggests that the experience of vection seems to be comparable between children and adults in some respects. In contrast, the latency or saturation of vection was significantly shorter or stronger in the children compared with the adults regardless of the difference in the visual motion pattern. This suggests that experienced vection can be more rapid or stronger in childhood than adulthood, as was also shown in previous developmental research (Shirai et al., 2012, 2014). Therefore, although the overall trend in vection seems to change over the lifespan (possibly beyond later childhood), the directional asymmetry in vection may emerge during an earlier stage of our lives (potentially early school age or younger).

Future research should investigate the development of other asymmetries in vection with a variety of directional patterns, such as upward versus downward motion or horizontal versus vertical motion. In addition, it would be interesting to examine the asymmetries in vection in older individuals. Haibach, Slobounov, and Newell (2009) reported that the saturation of vection is stronger in younger (mean age = 18.5 years) than in older adults (mean age = 64.9 and 75.0 years). Determining whether the asymmetries in vection are weaker in older adults as the saturation of vection will be a fruitful task to understand the entire developmental process of vection over the lifespan.
